# Analysis of a novel class A β-lactamase OKP-B-6 of *Klebsiella quasipneumoniae*: structural characterisation and interaction with commercially available drugs

**DOI:** 10.1590/0074-02760220102

**Published:** 2022-09-23

**Authors:** Reinaldo Bellini, Isabella Alvim Guedes, Luciane Prioli Ciapina, Ana Tereza Ribeiro de Vasconcelos, Laurent Emmanuel Dardenne, Marisa Fabiana Nicolás

**Affiliations:** 1 Laboratório Nacional de Computação Científica, Petrópolis, RJ, Brasil

**Keywords:** class A β-lactamase, OKP-B-6, multitarget antibiotics, Klebsiella quasipneumoniae, structure-based drug design, molecular docking

## Abstract

**BACKGROUND:**

Gram-negative and Gram-positive bacteria produce beta-lactamase as factors to overcome beta-lactam antibiotics, causing their hydrolysis and impaired antimicrobial action. Class A beta-lactamase contains the chromosomal sulfhydryl reagent variable (SHV, point mutation variants of SHV-1), LEN (*Klebsiella pneumoniae* strain LEN-1), and other *K. pneumoniae* beta-lactamase (OKP) found mostly in Klebsiella’s phylogroups. The SHV known as extended-spectrum β-lactamase can inactivate most beta-lactam antibiotics. Class A also includes the worrisome plasmid-encoded *Klebsiella pneumoniae* carbapenemase (KPC-2), a carbapenemase that can inactivate most beta-lactam antibiotics, carbapenems, and some beta-lactamase inhibitors.

**OBJECTIVES:**

So far, there is no 3D crystal structure for OKP-B, so our goal was to perform structural characterisation and molecular docking studies of this new enzyme.

**METHODS:**

We applied a homology modelling method to build the OKP-B-6 structure, which was compared with SHV-1 and KPC-2 according to their electrostatic potentials at the active site. Using the DockThor-VS, we performed molecular docking of the SHV-1 inhibitors commercially available as sulbactam, tazobactam, and avibactam against the constructed model of OKP-B-6.

**FINDINGS:**

From the point of view of enzyme inhibition, our results indicate that OKP-B-6 should be an extended-spectrum beta-lactamase (ESBL) susceptible to the same drugs as SHV-1.

**MAIN CONCLUSIONS:**

This conclusion advantageously impacts the clinical control of the bacterial pathogens encoding OKP-B in their genome by using any effective, broad-spectrum, and multitarget inhibitor against SHV-containing bacteria.

Beta-lactamases are enzymes produced by Gram-negative and Gram-positive bacteria as an evolutionary response against naturally occurring beta-lactams and constitute a significant resistance factor against beta-lactam antibiotics to control bacterial infections. The beta-lactam antibiotics target the bacterial cell wall, leading to cell lysis and death. However, in the presence of beta-lactamases, the beta-lactam drug is hydrolysed and inactivated, thereby preventing it from reaching the cell wall target. A thriving pharmaceutical industry strategy has been the beta-lactamase inhibitors (BLIs) designed to overcome this beta-lactamase-mediated inactivation of beta-lactam drugs. Thus, while the BLI binds to the beta-lactamase and interrupts its function, it enables the bactericidal effect of the beta-lactam antibiotics against pathogenic bacteria.^1^

Structurally, beta-lactamases are similar to penicillin-binding proteins (PBPs).^2^ ^,^ ^3^ Indeed, both enzymes acquired deacylation machinery to inactivate or hydrolyse beta-lactam antibiotics and have an α-helix domain and a mixed α/β domain, being the active site localised near the amino terminus of the central α-helix.^4^ The Ambler classification recognises four classes of beta-lactamases, namely A-D.^2^ While classes A, C, and D are active-site serine beta-lactamases, class B are structurally unrelated metallo beta-lactamases that use one or two Zn^+2^ catalytically functional atoms for beta-lactam hydrolysis.^2^ ^,^ ^5^ Mainly, class A includes an alarming group of extended-spectrum beta-lactamases (ESBLs) that can inactivate most beta-lactam antibiotics and carbapenemases that inactivate even carbapenems and some BLIs yielding a variant enzyme designated “inhibitor-resistant”,^6^ making infections caused by ESBL-producing and carbapenemase-producing bacteria tough to treat. The most worrying carbapenemase is the carbapenem-hydrolysing enzyme *Klebsiella pneumoniae* carbapenemase (KPC-2) found in *Klebsiella* phylogroups and other enteric bacteria.^7^ ^,^ ^8^ ^,^ ^9^

To overcome the activity of the ESBLs and carbapenemases, pharmaceutical challenges have focused on either the new generation of beta-lactams less susceptible to cleavage (*i.e.*, fifth-generation cephalosporins and ceftolozane, fourth-generation carbapenems) or BLIs with better inhibitory activity (*i.e.*, avibactam and vaborbactam).^10^ In the context of small molecules inhibiting beta-lactamases, structure-based computational methods have been applied to provide a rationale for the design of selective and broad-spectrum BLIs.^11^ ^,^ ^12^ For example, all “first generation” BLIs (*e.g.*, clavulanic acid, sulbactam, and tazobactam) function as mechanism-based compounds that utilise various strategies to inactivate the beta-lactamase. The “first generation” BLIs can promote semi-stable intermediates to inhibit serine beta-lactamases by preventing the deacylation machinery of this enzyme, or these BLIs yield covalent modifications on either Ser130 or the catalytic Ser70 residue on the active site of beta-lactamases.^11^ ^,^ ^12^ ^,^ ^13^ ^,^ ^14^ ^,^ ^15^ In the class of “second generation” BLIs, *i.e.*, avibactam (NXL104), approved by the FDA in 2015 as a ceftazidime/avibactam formulation, it can form an acyl-enzyme complex with a serine residue at the beta-lactamase binding site. Interestingly, this BLI can be removed from the beta-lactamase via deacylation, yielding an intact liberated inhibitor molecule.^16^ However, the carbapenemase KPC-2, carrying some amino acid substitutions in the Ω loop of the enzyme, is even capable of slowly desulfating avibactam, triggering the production of inactive BLI fragments.^17^ Currently, for treating strains producing the KPC-2 variant conferring resistance to ceftazidime-avibactam, the meropenem-vaborbactam combination becomes a valuable agent.^18^ It is important to note that clavulanic acid and penicillanic acid sulfones, sulbactam, and tazobactam exhibit a similar spectrum of inhibition. These inhibitors are recognised to inhibit most class A beta-lactamases, including ESBLs, and serine carbapenemases to a lesser extent.^19^ On the other hand, avibactam has a broader spectrum of activity than clavulanic acid and the sulfone inhibitors, potently inhibiting class-A penicillinases, ESBLs, serine carbapenemases, class-C cephalosporinases and some class-D oxacillinases.^20^

Although most beta-lactamases described to date are plasmid-encoded enzymes associated with hospital outbreaks, like the KPCs that increasingly spread among clinical isolates,^21^ ^,^ ^22^ ^,^ ^23^ there are specific chromosomally encoded beta-lactamase genes also identified from clinical isolates.^9^ ^,^ ^24^ The major variants of a chromosomal beta-lactamase gene, like *bla*_SHV_, *bla*_LEN_, and *bla*_OKP_ (OKP for other *K. pneumoniae* beta-lactamase)^25^ evolved in parallel with the diversification of three *K. pneumoniae* phylogenetic groups, namely KpI (*K. pneumoniae*), KpII (*K. quasipneumoniae*), and KpIII (*K. variicola*), but without concomitant phenotypic changes in beta-lactam susceptibility.^24^ ^,^ ^26^ Usually, the beta-lactamases variants could be a distinctive feature of these *Klebsiella* species since sulfhydryl reagent variable (SHV), point mutation variants of SHV-1 is predominantly found in *K. pneumoniae*, OKP in *K. quasipneumoniae*, and LEN (*K. pneumoniae* strain LEN-1) in *K. variicola*.^9^ Concerning the *K. quasipneumoniae*, the first isolates were described in 2014, belonging to subspecies *K. quasipneumoniae* subsp. *quasipneumoniae* (KpII-A) and *K. quasipneumoniae* subsp. *similipneumoniae* (KpII-B), carrying the divergent genes *bla*_OKP-A_ and *bla*_OKP-B_ in their chromosomes.^25^ ^,^ ^26^ ^,^ ^27^ A few studies have reported isolates belonging to this new subspecies describing their antimicrobial resistance genes repertoires.^28^ ^,^ ^29^ ^,^ ^30^ ^,^ ^31^ In Brazil, our group described at the genomic level a novel multidrug-resistant (MDR) clinical isolate of *K. quasipneumoniae* subsp. *similipneumoniae*, carrying both the OKP-B-6 variant enzyme and the plasmid-encoded beta-lactamase KPC-2. The new isolate called *Kqps*142 was associated with a hospital outbreak in southeast Brazil, representing a significant concern in public health.^32^

Considering the medical importance of beta-lactamases, the structures of a considerable number of these enzymes were elucidated, even various in complex with approved BLIs (more than 5,370 beta-lactamase structures from Bacteria in protein data bank (PDB), http://www.rcsb.org/pdb/ accessed on March 02, 2022). Most of the beta-lactamase PDB entries contain structural data from the ESBL class A, such as TEM [named after the patient (Temoneira) infected with the first isolate expressing TEM-1], cefotaxime-hydrolysing beta-lactamase isolated in Munich (CTX-M), SHV, and the carbapenemase KPC-2. However, no structure is yet available in PDB for OKP variant enzymes. Regarding the emergence of a novel variant beta-lactamase, such as OKP-B-6 of *K. quasipneumoniae* subsp. *similipneumoniae Kqps*142, diverse approaches to gain structural insight into these new enzymes are critically required. Since no 3D crystal structure has been determined for OKP-B so far, our goal was to perform structural characterisation and molecular docking studies of this new variant enzyme through its interaction with commercial BLIs.

## MATERIALS AND METHODS

*Sequence availability* - We retrieved the OKP-B-6 sequence from the genome of *K. quasipneumoniae* subsp. *similipneumoniae* Kqps142 (NCBI Bioproject number PRJNA383559), annotated as an OKP-B-6 beta-lactamase corresponding to GenBank accession no. AVR37864.1 and locus tag KPC142_02249 (https://www.ncbi.nlm.nih.gov/protein/AVR37864.1). We confirmed the final annotation as an OKP-B-6 beta-lactamase by the best match reference hit found in *K. pneumoniae* by “The Comprehensive Antibiotic Resistance Database” (CARD) with E-value = 0.0, 99% identity, and 100% coverage, which is also in agreement with Fevre et al.^27^ In the text, the number of the amino acid residues from the template sequence will be mentioned in parenthesis when discussing about the OKP-B-6 sequence and structure.

*Signal peptide cleavage* - The β-lactamases are secreted proteins (preproteins) containing an N-terminal signal peptide (SP) essential for targeting the cytoplasmic membrane and the translocation of the preprotein across the membrane. Then, upon translocating the secretory protein, the SP is cleaved off.^33^ According to the post-translational modification (PTM), we removed the SP from the original OKP-B-6 amino acid sequence using the SignalP 4.1 server (www.cbs.dtu.dk/services/SignalP) to proceed with the molecular modelling experiments.

*Comparative modelling* - We used the Swiss-Model server to construct the molecular 3D model of OKP-B-6 (https://swissmodel.expasy.org/).^34^ The selection of the template structure was based on the amino acid sequence identity and the presence of ligands in the active site. After the generation of the tridimensional model, we verified the stereochemical qualities by MOLPROBITY^35^ ^,^ ^36^ (Available at http://molprobity.biochem.duke.edu), PROCHECK,^37^ ^,^ ^38^ and ERRAT^39^ (available at https://saves.mbi.ucla.edu/). We compared the catalytic site of OKP-B-6 with both SHV-1 (PDB code 1VM1)^40^ and KPC-2 (PDB code 4ZBE)^16^ structures according to their electrostatic potentials calculated using the PDB2PQR^41^ and the Adaptive Poisson Boltzmann Solver server (APBS) available at http://server.poissonboltzmann.org/. The potential surface of the structures was generated with the Pymol v.2.0.7^42^ using the CHARMM force field considering a probe radius of 1.4 Å. The sulbactam binding cavity was characterised by DogSiteScorer, an automated pocket detection and analysis tool that used a grid-based method and support vector machine (SVM) for druggability assessment based on the 3D structure of the target protein.^43^

**Fig. 1:**
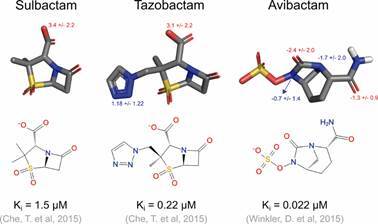
3D and 2D representation of the docked inhibitors with their experimentally measured binding affinities.^50^ ^,^ ^60^ The predicted pKa values of ionizable groups of sulbactam (CID 130313), tazobactam (CID 123630), and avibactam (CID 9835049) are labeled in red and blue.

*Preparation of the OKP-B-6 model and the structures of inhibitors* - We prepared the modeled structure of OKP-B-6 using the Protein Preparation Wizard tool from the Maestro suite (Schrödinger, LLC, New York, NY, 2020) considering two conserved water molecules in the binding site (Wat1 and Wat2, respectively numbered 636 and 656 in the 1VM1 template structure).^40^ These water molecules were considered since they are bind tightly at the binding site interacting with at least two amino acid residues, mediate hydrogen bonds between protein and ligand, and are conserved in other beta-lactamase structures [*e.g.*, PDB codes 4FH2 (SHV-1 S70C mutant complexed with sulbactam), 4ZAM (SHV-1 complexed with avibactam) and 4ZBE (KPC-2 complexed with avibactam)]. To predict the protonation states of the amino acid residues, we used PROPKA at pH = 7 and optimised the hydrogen bond network to adjust the orientation of the polar hydrogen atoms.^44^ According to the proposed mechanism provided by a recent study based on ultrahigh-resolution X-ray crystallography of the CTX-M-14 covalently bound to avibactam at different pH values, the *apo* form of class A β-lactamases has Lys48(73) positively charged and Glu141 (166) deprotonated,^45^ in agreement with previous studies.^46^ ^,^ ^47^ We also adopted this protonation state for Lys48 (73) and Glu141 (166) in OKP-B-6, which were predicted by PROPKA with pKa values of 9.48 and 4.95, respectively. The three-dimensional structures of the SHV-1 inhibitors tazobactam, sulbactam, and avibactam were retrieved from PubChem^48^ and prepared using Maestro to predict the protonation states and tautomers with Epik at pH 7.2 ± 0.2^49^ (Fig. 1).

*Docking methodology* - Molecular docking is a structure-based drug design strategy that aims to predict potential binding modes and the associated affinity of protein-ligand complexes, being extensively used to better understand the interaction profile between small molecules and therapeutic targets from many pathogens of clinical interest.^11^ ^,^ ^51^ ^,^ ^52^ ^,^ ^53^ ^,^ ^54^ In this work, we predicted the most favorable binding modes of the beta-lactamases inhibitors sulbactam, tazobactam, and avibactam against the constructed OKP-B-6 model through molecular docking with the DockThor program^55^ ^,^ ^56^ (freely available at www.dockthor.lncc.br). We compared the binding mode obtained for the OKP-B-sulbactam complex with the pre-acylation state of this inhibitor complexed with the Ser70Cys variant of SHV-1 (PDB code 4FH2)^57^ to validate the model constructed for OKP-B-6. The docking experiments were submitted using the standard configuration of the search algorithm: 1,000,000 evaluations, population size of 750, and 24 independent runs. The grid was centered on the sulbactam complexed with SHV1 in the pre-acylation state aligned to the OKP-B-6 model (X = 61.90, Y = 64.15, Z = 38.48) with a total size of 22 Å on each dimension and discretisation of 0.25 Å. We clustered the binding modes found using the *dtstatistic* tool from the DockThor platform. We ranked the top-energy binding modes according to the Total Energy (E_total_, the default DockThor scoring function for pose prediction composed of the torsional, van der Waals, and electrostatic terms from the MMFF94S force field).^58^ ^,^ ^59^

## RESULTS AND DISCUSSION

**Fig. 2:**
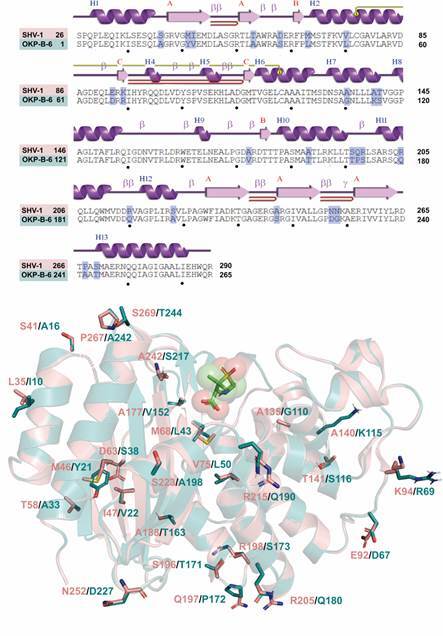
comparison of the 2D topology and 3D structure of other *Klebsiella pneumoniae* beta-lactamase (OKP-B-6) (coloured deep teal) andsulfhydryl reagent variable, point mutation variants of SHV-1 (coloured salmon). In the top is the alignment of sequences of the template SHV-1 and the OKP-B-6. Numbering in SHV-1 is according to the Ambler consensus for class A beta-lactamases, where the catalytic serine is set as Ser70. Non-identical residues are shaded in purple. The α-helices are depicted as H1 to H13. Turns are represented as β, and gamma turns are represented as γ. É highlights beta-hairpin. Strands are labeled as A, B, and C. The number 1 in yellow shows the disulfide bonds. Black spheres indicate the position at each 10 amino acid residues. Below is the structural alignment of the 3D structures of the template SHV-1 protein data bank (PDB) code 1VM1, coloured salmon and the constructed model of OKP-B-6 (coloured teal). The non-identical residues are represented in sticks. The structure of the pre-acylation state of sulbactam (PDB code 4FH2, aligned to the OKP-B-6 model) is represented as transparent spheres and sticks to highlight the binding site location.

*Comparative modelling* - Firstly, the SP sequence was removed, which was predicted at the N-terminal region from residues 1 to 21. Then, for the model building step, we considered the amino acid sequence from residues Ser22 to Arg286 as the mature chain of the OKP-B-6 enzyme mapped in the interval from 1 to 265 amino acids (Fig. 2A). The OKP-B-6 model was constructed using as template the crystal structure of SHV-1 beta-lactamase from *K. pneumoniae* inhibited by tazobactam (PDB code 1VM1, resolution of 2.02 Å and R-value work of 0.17),^40^ sharing 90.19% sequence identity. The Ramachandran plots confirmed good stereochemical quality, with 91.3% of residues in the most favored regions. The normalised QMEAN score is in the range [0.5,1.0], and the Z-score indicates values in typical ranges for similar proteins in the PDB with values of 0.22 ± 0.52. The ERRAT showed a quality factor of 94.16, comparable to other models obtained for beta-lactamases, where the value of ERRAT was 80.92.^39^ Due to the high identity of sequence with the SHV-1 template, the structure of OKP-B-6 conserved the secondary structures, composed of 13 α-helices (H1 to H13) and three β-sheets (A, B and C) (Fig. 2). The predicted protein structure is a monomer of 265 amino acids with one disulfide bond between residues Cys52 and Cys98, differing from the SHV-1 protein in only 25 residues that fall outside the binding pockets of interest.

**Fig. 3:**
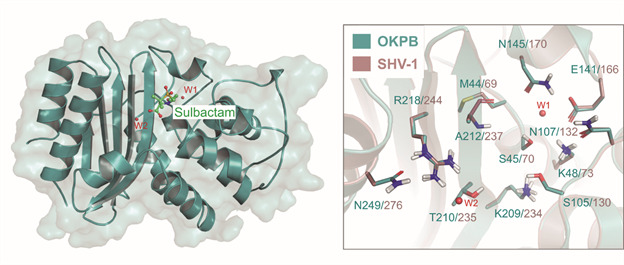
three-dimensional model of other *Klebsiella pneumoniae* beta-lactamase (OKP-B-6) (left) and comparison between OKP-B-6 and sulfhydryl reagent variable, point mutation variants of SHV-1 binding sites (right). Structural waters present are represented as red spheres. The sticks of main chains are hidden for clarity purposes, except for Ala212 (SHV-1 Ala237). The structure of the pre-acylation state of sulbactam protein data bank (PDB) code 4FH2, aligned to the OKP-B-6 model, highlights the binding site location.

As expected from the sequence alignment and structural similarity, the residues at the binding site are highly conserved between SHV-1 and OKP-B-6 (Figs 2-3), including the catalytically important residues Lys48, Ser105, and Glu141, suggesting that OKP-B-6 is also susceptible to the same substrate and inhibitors. Furthermore, the residues involved with beta-lactamases resistance are also conserved in OKP-B-6, mainly Met44, Ser105, Lys206, Arg218, and Asn249.^60^

**Fig. 4:**
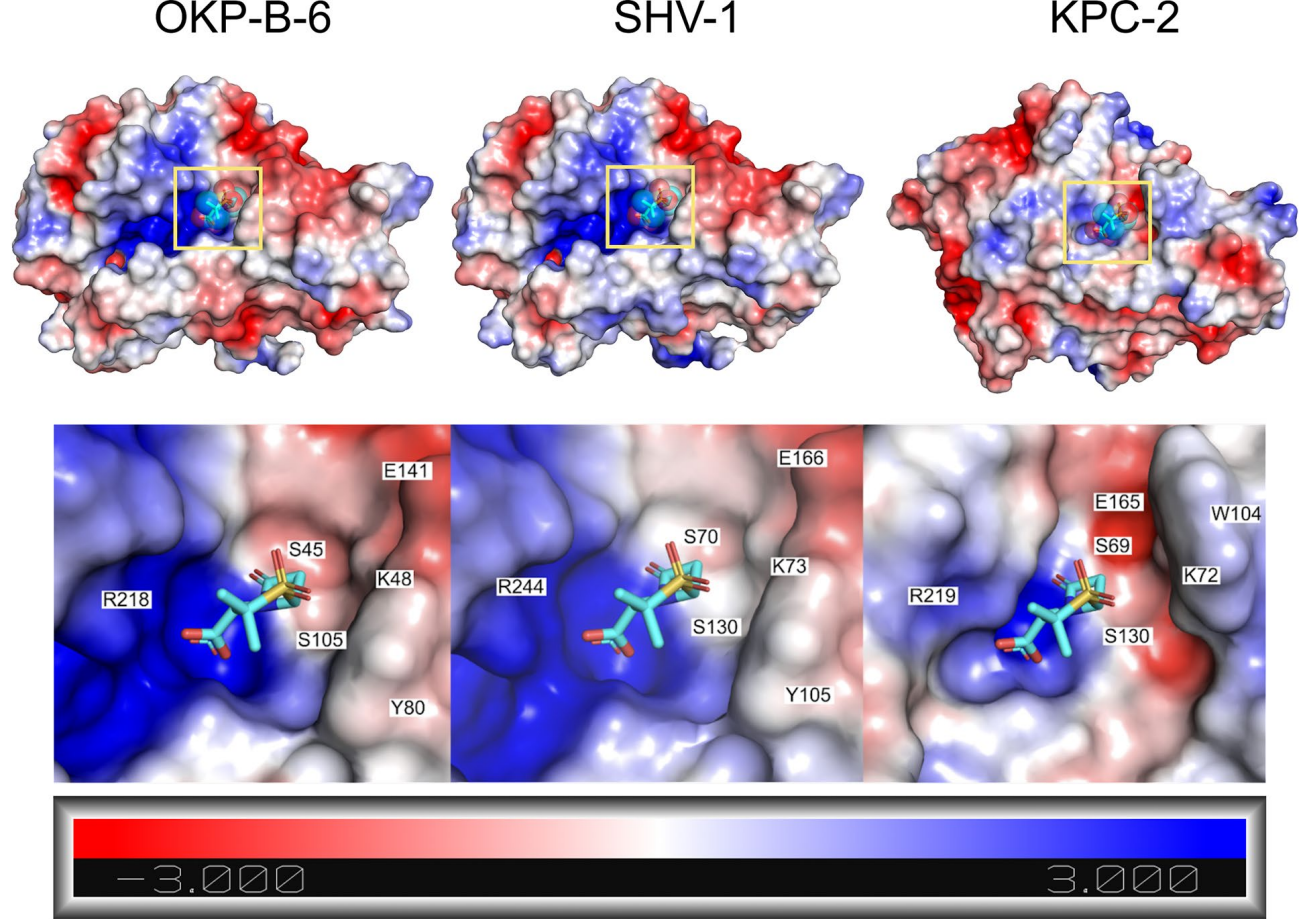
electrostatic potential of (A) other *Klebsiella pneumoniae* beta-lactamase (OKP-B-6), (B) sulfhydryl reagent variable, point mutation variants of SHV-1 and (C) *K. pneumoniae* carbapenemase (KPC-2) focusing on the sulbactam binding site. The electrostatic potential was calculated in Pymol/Adaptive Poisson-Boltzmann Solver (APBS) at pH 7, ranging from blue (+3 kT/e, positively charged) to red (-3 kT/e, negatively charged). The structure of the pre-acylation state of sulbactam protein data bank (PDB) code 4FH2, aligned to the OKP-B-6 model, is present to highlight the binding site location, but it was not present during the potential calculation.

**Fig. 5:**
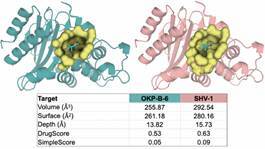
sulbactam binding cavity predicted with DogSiteScorer for other *Klebsiella pneumoniae* beta-lactamase (OKP-B-6) and sulfhydryl reagent variable, point mutation variants of SHV-1. DrugScore and SimpleScore range from 0 to 1, so the higher the score, the more druggable the pocket is estimated to be. The binding site location is highlighted by the structure of the pre-acylation state of sulbactam protein data bank (PDB) code code 4FH2,^52^ aligned to the OKP-B-6 model.

We also analysed the differences between the catalytic sites of OKP-B-6, SVH-1, and KPC-2 through the comparison of their electrostatic profiles and volumes (Fig. 4). All enzymes exhibited a positively charged environment in the carboxylate binding pocket, with OKP-B-6 and SVH-1 exhibiting a larger pocket with an electrostatic potential more pronounced than that of KPC-2 in this region. The positive charge on both catalytic sites is known to be critical for guiding the negatively charged substrates. In Fig. 5, the sulbactam binding cavity, as predicted with DogSiteScorer, indicates that this pocket is slightly smaller in OKP-B-6 than in the SHV-1 structure, with a volume of 255.87 Å^3^ (292.54 Å^3^ in SHV-1) and a surface of 261.18 Å^2^ (280.16 Å^2^ in SHV-1), and similar druggability scores of DrugScore = 0.53 (0.62 in SHV-1) and SimpleScore = 0.05 (0.09 in SHV-1).

*Docking of the inhibitors against OKP-B-6* - Sulbactam and tazobactam have similar mechanisms of action, inhibiting beta-lactamases through a serine-acylation mechanism.^15^ ^,^ ^57^ Avibactam, an inhibitor that has the beta-lactam core replaced by a 5-membered ring with a carbonyl bond adjacent to ring nitrogen, has been proposed to inhibit the enzyme through a distinct mechanism of action, despite interacting with the same key residues within the binding site.^12^ ^,^ ^16^

**Fig. 6:**
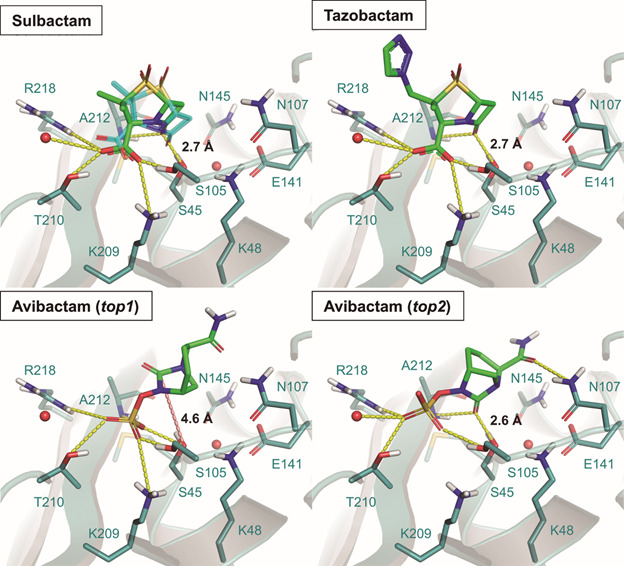
docking results for the inhibitors sulbactam, tazobactam, and avibactam against the other *Klebsiella pneumoniae* beta-lactamase (OKP-B-6) model. The pre-acylation state of sulbactam observed experimentally protein data bank (PDB) code 4FH2 is represented as cyan sticks. Conserved waters are represented as red spheres. Hydrogen bonds are represented as yellow dashes, and the distance between the carbonyl oxygen and Ser45 is highlighted. Salmon dashed line represents the distance between the carbonyl carbon atom of avibactam (top-1 pose) and Ser45 hydroxyl oxygen.

Our docking protocol against OKP-B-6 successfully predicted the experimental binding mode of sulbactam observed in the pre-acylation state of SHV-1-sulbactam (PDB code 4FH2) with an RMSD of 0.97 Å (E_total_ = -21.88 kcal/mol), which is also similar to the binding mode proposed for the recognition of beta-lactam substrates (PDB code 1KVL)^57^ ^,^ ^61^ (Fig. 6 and Table). After the acylation step, the ligand undergoes a significantly conformational change,^61^ as observed in many experimentally solved structures of the acyl-enzyme intermediates for different inhibitors. Since sulbactam and tazobactam have the same mechanism of action, it is expected that their binding modes are similar before the covalent bond formation. Despite no experimental structure of the pre-acylation state of tazobactam is available, its top-ranked predicted pose (E_total_ = -31.03 kcal/mol) perfectly fits the experimental binding mode of sulbactam, being characterised by the key interactions with the binding site, namely: (i) hydrogen bonds from the carbonyl oxygen from the beta-lactam ring with the oxyanion hole formed by Ala212 (237) and the catalytic Ser45 (70), (ii) the stabilisation of the negatively charged carboxyl moiety by strong hydrogen bonds with Thr210 (235), Wat2 and the catalytic Ser105 (130), and through salt bridges with Arg218 (244) and Lys209 (234) (Fig. 6). The top-ranked docked pose of avibactam (E_total_ = -22.91 kcal/mol, Fig. 6 and Table) is characterised by the positioning of the sulfate moiety at the carboxyl binding pocket, interacting with Ser45 (70), Ser105 (130), Thr210 (235) and Arg218 (244) through hydrogen bonds, whereas the carbonyl oxygen from the terminal amide group is oriented to the side chains of Asn107 (132) and Asn145 (170). However, in this binding mode, the carbonyl carbon atom of the 5-membered ring, expected to suffer the nucleophilic attack, is exposed to the solvent, opposed to the Ser45 (70) (4.6 Å). Exploring the different binding modes predicted by DockThor, we found that the top-2 best-docked conformation of avibactam (E_total_ = -19.10 kcal/mol) is more likely to be the pre-acylation state, with the carbonyl oxygen atom located at the oxyanion hole interacting with the side chain of Ser45 (70) at 2.6 Å and the main chain of Ala212 (237), as observed in the experimental structures of avibactam complexed with different beta-lactamases.^16^ ^,^ ^46^ ^,^ ^62^ ^,^ ^63^ ^,^ ^64^ The docking results obtained in this work for the evaluated SHV-1 inhibitors against the OKP-B-6 binding site, associated with the highly conserved residues when compared to the SHV-1 target, suggest that OKP-B-6 is susceptible to the same drugs.

**TABLE:** Docking results of the inhibitors sulbactam, tazobactam and avibactam against the other *Klebsiella pneumoniae* beta-lactamase (OKP-B-6) model. The two top-ranked binding modes of avibactam were selected according to the total energy, which is the default scoring function for pose prediction in the DockThor program

Compound	Total energy^*a*^	Elec. energy^*a*^	vdW energy^*a*^
Sulbactam	-21.88 (0.97^*b*^ )	-39.70	1.88
Tazobactam	-31.03	-39.00	-0.40
*Avibactam* (top-1)	-22.91	-38.16	-4.32
*Avibactam* (top-2)	-19.10	-26.53	-11.39

*a*: given in kcal/mol; *b*: root-mean-square deviation from the pre-acylation state of sulbactam protein data bank (PDB) code 4FH2, given in Å.

*In conclusion* - Our results indicate that OKP-B-6 should be an ESBL like SHV-1, with both beta-lactamases having the amino acid residues within the binding cavities highly conserved. We identified that the predicted binding modes with the docking of either sulbactam, tazobactam, or avibactam with OKP-B-6 are similar to those observed experimentally with SHV-1, which is expected since these inhibitors have broad activity against equivalent ESBLs. The docking results obtained in this work validated the constructed OKP-B-6 model and provided useful insights for its inhibition. These results also have important implications for clinical microbiologists in the control of pathogens that carry one of these types of class A beta-lactamases, SHV, or OKP-B. Particularly, the formulation prescribed with one of the inhibitors tested here would also be effective in controlling *K. quasipneumoniae* carrying OKP-B.
